# Ocean Aerobiology

**DOI:** 10.3389/fmicb.2021.764178

**Published:** 2021-10-29

**Authors:** Alyssa N. Alsante, Daniel C. O. Thornton, Sarah D. Brooks

**Affiliations:** ^1^Department of Oceanography, Texas A&M University, College Station, TX, United States; ^2^Department of Atmospheric Sciences, Texas A&M University, College Station, TX, United States

**Keywords:** aerobiota, biogenic aerosol, microbial oceanography, cloud condensation nuclei (CCN), ice nucleating particles (INPs), sea spray aerosol (SSA), atmospheric dispersal, air-sea interaction

## Abstract

Ocean aerobiology is defined here as the study of biological particles of marine origin, including living organisms, present in the atmosphere and their role in ecological, biogeochemical, and climate processes. Hundreds of trillions of microorganisms are exchanged between ocean and atmosphere daily. Within a few days, tropospheric transport potentially disperses microorganisms over continents and between oceans. There is a need to better identify and quantify marine aerobiota, characterize the time spans and distances of marine microorganisms’ atmospheric transport, and determine whether microorganisms acclimate to atmospheric conditions and remain viable, or even grow. Exploring the atmosphere as a microbial habitat is fundamental for understanding the consequences of dispersal and will expand our knowledge of biodiversity, biogeography, and ecosystem connectivity across different marine environments. Marine organic matter is chemically transformed in the atmosphere, including remineralization back to CO_2_. The magnitude of these transformations is insignificant in the context of the annual marine carbon cycle, but may be a significant sink for marine recalcitrant organic matter over long (∼10^4^ years) timescales. In addition, organic matter in sea spray aerosol plays a significant role in the Earth’s radiative budget by scattering solar radiation, and indirectly by affecting cloud properties. Marine organic matter is generally a poor source of cloud condensation nuclei (CCN), but a significant source of ice nucleating particles (INPs), affecting the formation of mixed-phase and ice clouds. This review will show that marine biogenic aerosol plays an impactful, but poorly constrained, role in marine ecosystems, biogeochemical processes, and the Earth’s climate system. Further work is needed to characterize the connectivity and feedbacks between the atmosphere and ocean ecosystems in order to integrate this complexity into Earth System models, facilitating future climate and biogeochemical predictions.

## Introduction

The origins of ocean aerobiology can be traced to Darwin (1846), who reported that ships in the Atlantic Ocean often became coated in a layer of fine dust, thought to have originated in Africa. Darwin collaborated with Christian Gottfried Ehrenberg (a pioneer of microscopy) to study the content of dust he himself collected on the *HMS Beagle* in 1833, and samples collected by sailors on other ships. Ehrenberg identified 67 taxa of “infusoria” (protists), including two marine taxa (Darwin, 1846). Despite early scientific interest in aerobiology, recent estimates of the abundance and biomass of organisms on Earth have ignored the atmosphere (Whitman et al., 1998; Bar-On et al., 2018) or considered the atmosphere to be a minor component (Flemming and Wuertz, 2019). Certainly, the biomass and abundance of microorganisms in the atmosphere (5 × 10^22^ prokaryotes; Flemming and Wuertz, 2019) is low compared with other environments (1 × 10^29^ prokaryotes in the ocean; Flemming and Wuertz, 2019), but low abundance does not preclude significance.

**FIGURE 1:**
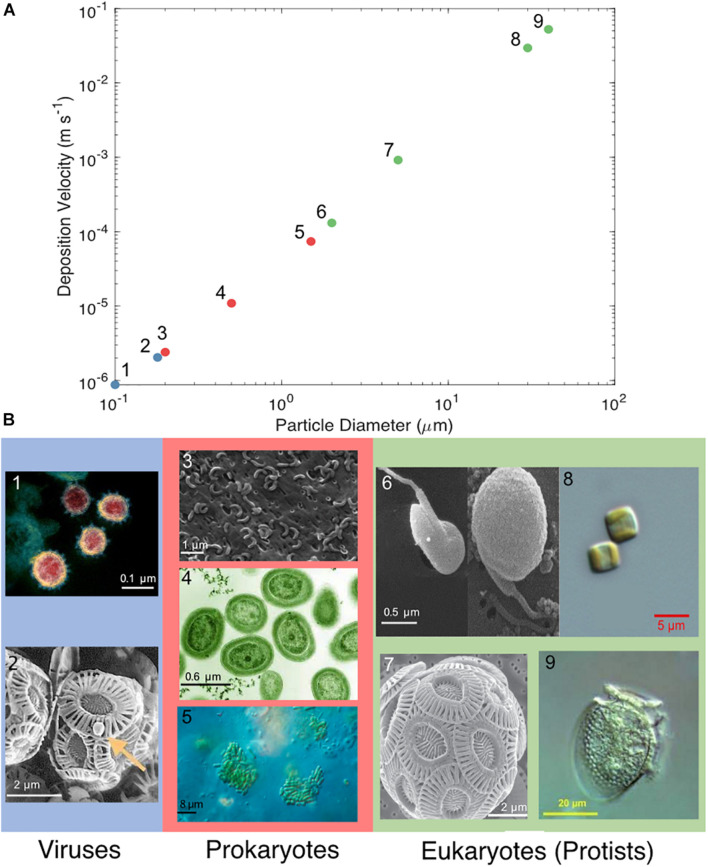
**(A)** Deposition velocity of varying cell diameters of viruses (blue), prokaryotes (red), and eukaryotes (green). **(B)** Microscopy images of microorganisms representative of the particle diameter chosen: (1) SARS-CoV-2, (2) *Emiliania huxleyi* virus, (3) SAR11, (4) *Prochlorococcus*, (5) *Synechococcus*, (6) *Micromonas pusilla*, (7) *Emiliania huxleyi*, (8) *Thalassiosira* sp., and (9) *Dinophysis acuminata*. (1–7) Approximate scale bars were added. The microscopy images in **(B)** were reproduced under the Creative Commons Attribution International licenses and were captured by (1) NIAID’s Rocky Mountain Laboratories in Hamilton, Montana (2) Wikimedia commons (3) Steindler et al. (2011) (4) Luke Thompson at the Sallie Chisholm Lab and Nikki Watson at Whitehead, MIT (5) Proyecto Agua at Biodiversidad virtual, Cantabria, Spain (6) Manton and Parke (1960) and courtesy of Nordic Microalgae and Aquatic Protozoa (Karlson et al., 2020) (7) Alison R. Taylor, University of North Carolina Wilmington Microscopy Facility (8) Moore et al. (2017) and (9) Plankton Net, Alfred Wegener Institute, Helmholtz Centre for Polar and Marine Research.

**FIGURE 2:**
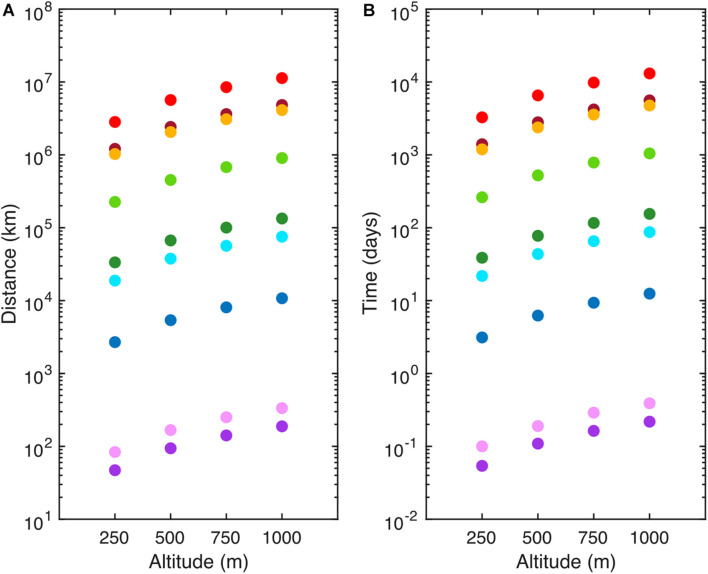
Atmospheric transportation of representative marine microorganisms and Coronavirus (SARS-CoV-2) **(A)** Potential distance traveled (km) and **(B)** residence time (days) of SARS-CoV-2 (red), *Emiliania huxleyi* virus (maroon), SAR11 (orange), *Prochlorococcus* (light green), *Synechococcus* (dark green), *Micromonas pusilla* (light blue), *Emiliania huxleyi* (dark blue), *Thalassiosira weissflogii* (light purple), and *Dinophysis acuminata* (dark purple). Values were determined using Stoke’s Law for aerosol particles released at specific altitudes (250–1,000 m) at the average tropospheric temperature (15°C) in a homogeneous atmosphere. Residence time was calculated using a representative average wind speed of 10 m s^–1^. Distance traveled and residence time should be considered the upper limit as these estimates do not account for loss processes such as wet deposition.

**FIGURE 3:**
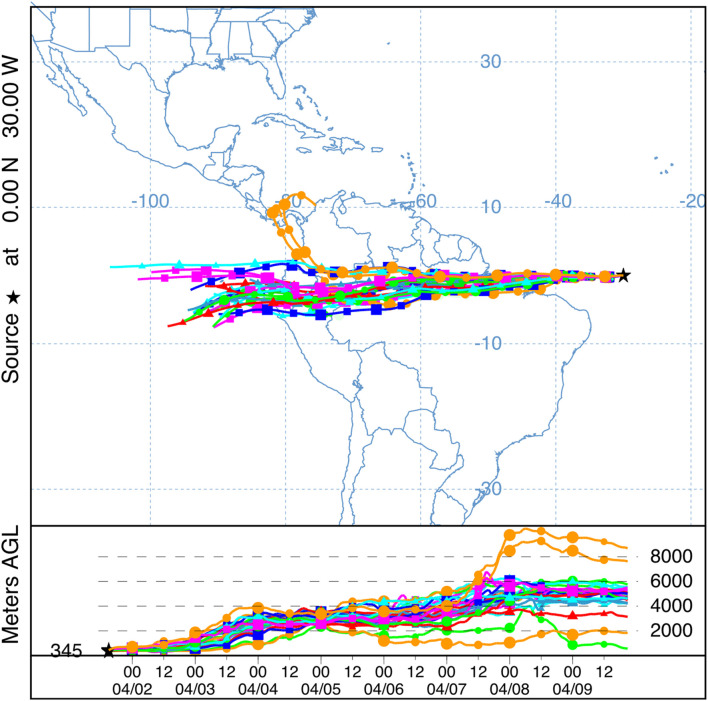
An airmass traveling from the Atlantic to the Pacific was tracked using NOAA Air Resources Laboratory HYSPLIT (Stein et al., 2015; Rolph et al., 2017) forward trajectory ensemble model with a starting point at 0°N, 30°W (black star) over 7 days (04/02/2020 –04/09/2020) with global forecast system (GFS) meteorology data. Each possible trajectory is represented with each point along the trajectory corresponding to a 12-h increment. The estimated mid-boundary layer height was added (345 m) at the starting point (black star) and the height (m) above ground level (AGL) is shown for each trajectory. Multiple colors enables individual trajectories to be visualized.

Airborne marine microorganisms affect the distribution of specific taxa in the ocean (Womack et al., 2010), ecosystem structure, and genetic exchange between ecosystems. Marine microorganisms are extremely diverse, as exemplified by their range of shapes and sizes (Figure 1). Size plays a major role in determining the deposition velocity of airborne microorganisms (Figure 1). Deposition velocity varies over five orders of magnitude for typical marine microorganisms, with lows of 9 × 10^–7^ m s^–1^ for viruses and higher values of 5 × 10^–2^ m s^–1^ for large eukaryotes (Figure 1). Therefore, a marine microorganism’s size strongly influences potential distance transported and residence time in the atmosphere (Figure 2). Atmospheric transport potentially carries viable marine microorganisms between locations that are inaccessible via surface currents due to geographical barriers and relatively long timescales (Figures 2, 3). For example, a parcel of water in the Atlantic can take more than 9 years to travel to the Pacific by surface currents (Jönsson and Watson, 2016), whereas atmospheric dispersal between Atlantic and Pacific occurs over a few days (Figure 3). The significance of aerial transport of marine microorganisms depends on three factors: (1) the fluxes of organisms between ocean and atmosphere; (2) how far marine microorganisms are transported in the atmosphere; and, (3) what proportion of marine microorganisms are viable after deposition in the ocean.

Air-sea exchange of microbial communities is dynamic, with hundreds of trillions of microorganisms emitted to and deposited from the atmosphere daily (Mayol et al., 2014). Mayol et al. (2017) estimated total emission fluxes of prokaryotes (1 × 10^3^ to 2 × 10^6^ cells m^–2^ day^–1^) and eukaryotes (1–2000 cells m^–2^ day^–1^) over the tropical and subtropical ocean. The estimated deposition fluxes were up to 6 × 10^6^ prokaryotes m^–2^ day^–1^ and 2 × 10^7^ eukaryotes m^–2^ day^–1^ over tropical and sub-tropical regions with nearby land masses, indicating terrestrial sources dominate the aerobiota over the ocean in many locations (Mayol et al., 2017). However, the fraction of terrestrial microbial species decreases with distance from land and between 33 and 68% of the microorganisms over the ocean are likely to be of marine origin (Mayol et al., 2017). A lack of spatial and temporal sampling density limits our understanding of atmospheric abundance and biodiversity. There is currently only one study of global airborne microbial communities, and it does not include pristine marine sampling sites (Tignat-Perrier et al., 2019).

This review will explore the composition and formation of biogenic marine aerosol. Secondly, we will review the significance of marine aerosol to ecosystem processes and structure, considering the transport and viability of marine microorganisms in the atmosphere, their biogeographical relevance, and potential to influence marine ecosystem processes and biogeochemistry. Finally, we will review the role of marine biogenic aerosols in weather and climate (DeMott et al., 2016; Brooks and Thornton, 2018; Croft et al., 2021), particularly the role of marine primary aerosol in the natural seeding of clouds (Wilbourn et al., 2020; Hendrickson et al., 2021; Zheng et al., 2021). We will synthesize existing knowledge and suggest future research directions to gain a better understanding of the sources, fate, and significance of marine organic aerosol.

## Generation of Marine Aerosol

**FIGURE 4:**
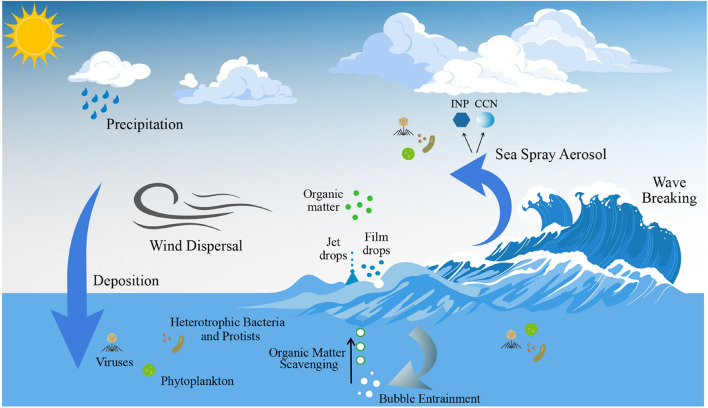
Physical processes associated with the generation of sea spray aerosol (SSA). Breaking waves entrain air, which results in bubbles bursting at the air-sea interface, lofting jet and film drops into the atmosphere, which form SSA. A subset of SSA catalyzes cloud formation by acting as cloud condensation nuclei (CCN) or ice nucleating particles (INPs). Primary marine aerosol formation via sea spray results in the transfer of marine microorganisms and organic matter to the atmosphere, which are transported over the ocean and deposited in a new location via processes such as wet deposition. The vector art used in this figure was downloaded from vecteezy.com.

Primary marine aerosol are directly ejected into the atmosphere from the ocean surface (Quinn et al., 2014, 2015; Brooks and Thornton, 2018). Wave breaking causes entrainment of air bubbles in the underlying water, which scavenge organic matter as they rise to the surface and burst (Blanchard and Woodcock, 1957; Blanchard, 1963; Gantt and Meskhidze, 2013; Figure 4). Bubble bursting produces two types of primary sea spray aerosol (SSA), film and jet drops, both containing a mixture of sea salt and marine organics (Quinn et al., 2015; Wang et al., 2017). Film drops form due to the fragmentation of the film cap surrounding the bubble, and jet drops are produced when the film cap disintegrates, causing the collapse of the bubble cavity. Jet drop formation typically results in SSA that is supermicrometer in size and contains mainly sea salt and water-soluble organic matter (Wang et al., 2017), as well as larger microorganisms. Film drops are submicrometer (Veron, 2015), comprising hydrophobic organic matter (Wang et al., 2017) and likely small cellular microorganisms (e.g., bacteria) and cell fragments, as well as viral particles (Blanchard and Syzdek, 1982; Rastelli et al., 2017; Michaud et al., 2018). Organic matter is enriched in SSA compared to underlying water in biologically active areas of the ocean (O’Dowd et al., 2004; Gantt et al., 2011). Although significant seasonal variations have been observed (Sanchez et al., 2018; Quinn et al., 2019; Saliba et al., 2020), concentrations of marine aerosol are relatively low (generally less than 500 cm^–3^), compared with 1,000–2,000 cm^–3^ over continental landmasses (Wallace and Hobbs, 2006). Nevertheless, given the vast area for air-sea exchange (362 million km^2^, or 71%, of the Earth’s surface; Charette and Smith, 2010), marine primary aerosols play a significant role in Earth system processes.

At wind speeds up to 10–13 m s^–1^, the ocean is covered in gelatinous “skin” known as the sea surface microlayer (SML) (Salter et al., 2011; Wurl et al., 2011; Sabbaghzadeh et al., 2017). The SML is operationally defined as the top 1–1,000 μm of the ocean’s surface, consisting of a unique environment at the air-sea interface with distinct biological, chemical, and physical properties (Liss and Duce, 1997). Organic matter can become up to 1,000 times enriched in the SML compared to the underlying water (Liss and Duce, 1997) and include surface active proteins, lipids, and carbohydrates, as well as protists, bacteria, and viruses (Leck and Bigg, 2005; Orellana et al., 2011; Cunliffe et al., 2013; Engel et al., 2017). The SML contains a distinctive microbial community composition compared with the underlying water (Franklin et al., 2005; Joux et al., 2006; Cunliffe et al., 2009, 2011; Stolle et al., 2011). Although the exact controls determining the microbial community within the SML are not known, these microorganisms have been shown to withstand meteorological influences, such as increased turbulence and breaking waves (Stolle et al., 2011), and ultraviolet light (Ortega-Retuerta et al., 2009; Santos et al., 2011). Viruses have been overlooked, but viral lysis of cells may be a significant determinant of organic matter enrichment and microbial composition in the SML, and subsequently the composition of organic matter in primary marine aerosol (Rahlff, 2019). Microorganisms become embedded and enriched within a gel matrix of polymeric surfactants in the SML (Aller et al., 2005; Cunliffe and Murrell, 2009; Thornton et al., 2016), such as transparent exopolymer particles (TEP) (Alldredge et al., 1993) and Coomassie staining particles (CSP) (Long and Azam, 1996). This is consistent with the identification of polysaccharides in marine aerosol from the North Atlantic Ocean (Aller et al., 2017; Lawler et al., 2020). It has been presumed that the SML plays a dominant role in determining the composition of the organic matter is SSA. However, recent observations suggest that the direct contribution to SSA from the SML is minor compared with bubble plumes from the underlying water (Chingin et al., 2018; Frossard et al., 2019). Bubble plumes cause the SML to become displaced and therefore remove SML organics from the pathway of bubble bursting at the ocean’s surface (Long et al., 2014). Consequently, the concentration of organic matter in rising bubbles from the subsurface is proportional to the concentration of organic matter observed in the atmosphere via bubble bursting (Tseng et al., 1992).

While primary aerosol are the focus of this review, the significance of secondary aerosol that form in the atmosphere from gaseous precursors must be mentioned for a complete overview of marine biogenic aerosol. Secondary aerosols are produced via the oxidation of volatile organic compounds (VOCs) emitted by phytoplankton and bacteria (Halsey et al., 2017; Davie-Martin et al., 2020; Fox et al., 2020; Moore et al., 2020; Croft et al., 2021; Zheng et al., 2021), as well as photochemical reactions in the SML (Bernard et al., 2016; Brüggemann et al., 2018). Marine VOCs include acetaldehyde, acetone, acetonitrile, dimethyl sulfide (DMS), isoprene, methanethiol, methanol isoprene and halocarbons (Shaw et al., 2003; Dixon et al., 2013; Liu et al., 2013a, b; Halsey et al., 2017; Davie-Martin et al., 2020). DMS is a source of secondary aerosol in the form of non-sea salt sulfates, which is proposed as a significant source of cloud condensation nuclei (CCN) (Charlson et al., 1987; Gantt and Meskhidze, 2013; Quinn et al., 2019). New particle formation from the oxidation of VOCs in the upper marine boundary (Croft et al., 2021; Zheng et al., 2021) is significant, though poorly constrained.

Our understanding of links between seawater properties and overlying aerosol are tenuous (Saliba et al., 2019) due to the range of timescales and complexity of processes occurring in both the ocean and atmosphere. In the ocean, properties such as sea surface temperature, salinity, and surfactant concentration affect the rise time of bubbles and the bubble-burst processes (Fuentes et al., 2010; Forestieri et al., 2018; Saliba et al., 2019). Biological processes affect the composition and concentration of the organic matter available for aerosolization (O’Dowd et al., 2004, 2015; Fuentes et al., 2010; Rinaldi et al., 2013). Once in atmosphere, transport, mixing, and photochemical processing affect the composition of the aerosol (Saliba et al., 2019). The influence of wind speed on the formation and processing of aerosols remains one of the major challenges (Saliba et al., 2019, 2020). Supplementary Tables 1, 2 summarizes global emission estimates and measured concentrations of both organic carbon and sea salt emitted as SSA.

## Organic Matter Contributions to Primary Marine Aerosol

Nascent SSA has been linked to short-lived, labile, forms of DOM using sea surface chlorophyll-*a* concentrations as an indicator of phytoplankton primary production and ecosystem activity (O’Dowd et al., 2004; Fuentes et al., 2010; Rinaldi et al., 2013). Other studies attribute DOM in SSA to older, recalcitrant sources, indicating that the enrichment of SSA with DOM is uncoupled from phytoplankton growth on seasonal and shorter timescales (Bates et al., 2012, 2020; Quinn et al., 2014; Kieber et al., 2016; Beaupré et al., 2019). Because the lifetime of recalcitrant DOM is much longer than the average mixing time of the ocean (Hansell et al., 2012), recalcitrant DOM is well mixed, forming a relatively uniform aerosol source. Understanding the chemical composition and origin of organic matter in SSA is key for incorporating SSA into climate models. SSA dominated by recalcitrant organic matter could be represented by a series of constant and global parameters, whereas a dynamic pool of organic matter in SSA will require complex modeling accounting for ecosystem processes and continuous environmental change, such as the seasons.

While the DOM inventory of the ocean (662 Pg C; Hansell et al., 2009) is dominated by recalcitrant organic matter (>612 Pg C; Hansell, 2013), the major source of labile organic matter is photosynthetic productivity, which is generally associated with sunlit surface waters. Therefore, it is likely that nascent SSA generally contains a complex mixture of DOM from labile to ultra-recalcitrant. Chemical characterization of samples from the North Atlantic by Lawler et al. (2020) supports this hypothesis. Lawler et al. (2020) concluded that there was a seasonal signal associated with labile polysaccharides and a relatively constant pool of recalcitrant organic matter associated with alcohol groups. Strictly speaking, the thermally stable (i.e., refractory) DOM identified by Lawler et al. (2020) is not necessarily equivalent to the recalcitrant pool defined by Hansell (2013). Current hypotheses explaining the stability of recalcitrant DOM are based on unavailability to microorganisms rather than thermal stability (Hansell, 2013).

The major classes of organic compounds in SSA reflects the composition of living organisms and non-living organic matter processed through the microbial food web (Table 1). Processes such as bubble bursting are selective and fractionate DOM across the air-sea interface according to chemical properties, such as surface tension, solubility, and interactions with ions (Cochran et al., 2017). Consequently, the organic composition of SSA is not identical to the available pool of organic matter in the underlying water (Brooks and Thornton, 2018; Table 1). Many compounds are significantly enriched in SSA concentration compared to the underlying seawater (Table 1). Chemical components of SSA should be explored for biomarkers to link the composition of SSA to specific organisms or biogeochemical processes in the originating waters. Chromophoric and fluorescent DOM (Yamashita and Tanoue, 2008), and some proteins (Aluwihare et al., 2005; Table 2), have the potential to be used as biomarkers for recalcitrant DOM. Monosaccharides are present in primary marine aerosol (Russell et al., 2010; Frossard et al., 2014; Zeppenfeld et al., 2021) and could serve as a biomarker for recent biological activity as they are labile (Table 2). Labile organic matter also includes mono- and dicarboxylic acids, fatty acids, lipids, and glycerols (Mochida et al., 2002; Wang et al., 2015; Cochran et al., 2016, 2017; Moran et al., 2016; Rastelli et al., 2017). Rastelli et al. (2017) provided the first indication of extracellular DNA enrichment in marine aerosol samples and hypothesized processes leading to cell lysis, such as viral infection, led to the presence of nucleic acid found in SSA. Amino acids and proteins are enriched in primary aerosol (Kuznetsova et al., 2005; Hawkins and Russell, 2010; Rastelli et al., 2017; Schiffer et al., 2018). Proteins in the form of microbial enzymes (e.g., lipase, protease, and alkaline phosphatase) are a component of marine aerosol (Wang et al., 2017; Schiffer et al., 2018; Malfatti et al., 2019). Some enzymes have a higher activity in SSA when compared to subsurface water and may modify aerosol droplet chemistry and physical properties (Malfatti et al., 2019). Enzymes in the atmosphere potentially impact radiative budgets and aerosol-cloud interactions by changing the composition and surface properties of aerosol.

**TABLE 1:** Organic compounds observed in marine aerosol.

Class of compound		Functional group	Enrichment in SSA	References
Fatty Acids	Lipids Fatty acids	Carboxyl	Factor of 140,000 (Rastelli et al., 2017) Detected	Mochida et al., 2002; Schmitt-Kopplin et al., 2012; Wang et al., 2015; Cochran et al., 2016, 2017; Rastelli et al., 2017
Carbohydrates	Monosaccharides Polysaccharides	Carbonyl Hydroxyl	Factor of 669 ± 143 Factor of 10,000	Facchini et al., 2010; Hawkins and Russell, 2010; Gao et al., 2012; Fu et al., 2013; Jayarathne et al., 2016; Cochran et al., 2017; Rastelli et al., 2017
	Exopolymer particles	Carbonyl Hydroxyl	Factor of 100–1,000	Aller et al., 2005, 2017; Leck and Bigg, 2005; Orellana et al., 2011; Burrows et al., 2013
Proteins	Amino acids triacylglycerol lipase	Amine Carboxyl	Factor of 120,000 (Total proteins) (Rastelli et al., 2017)	Kuznetsova et al., 2005; Hawkins and Russell, 2010; Rastelli et al., 2017; Schiffer et al., 2018
Nucleic Acid	DNA RNA	Amine Carbonyl Hydroxyl Phosphate	Factor of 30,000 (DNA) (Rastelli et al., 2017)	Cho and Hwang, 2011; Sharoni et al., 2015; Rastelli et al., 2017; Michaud et al., 2018; Amato et al., 2019
Pigments	Chlorophyll-*a*	Carbonyl Methyl	Detected	O’Dowd et al., 2004, 2008; Yoon et al., 2007; Fuentes et al., 2010; Vignati et al., 2010; Rinaldi et al., 2013; Long et al., 2014; Quinn et al., 2014

**TABLE 2:** Potential biomarkers of different sources of organic matter and microorganisms in the atmosphere.

Biomarker	Source	References
Muramic acid	Peptidoglycan and bacterial biomass	Zeppenfeld et al., 2021
3-hydroxy fatty acid	Bacterial biomass (gram-negative)	Bikkina et al., 2019
Ergosterol	Fungi	Burshtein et al., 2011
Polyols (mannitol, arabitol)	Fungi	Bauer et al., 2008; Tignat-Perrier et al., 2019
Chlorophyll and Chlorophyllide *a*	Phytoplankton	Miyazaki et al., 2020
Silica	Diatoms	Cochran et al., 2017; McCluskey et al., 2018
Calcium carbonate	Coccolithophores	Hawkins and Russell, 2010; Trainic et al., 2018
Chromophoric DOM	Recalcitrant DOM	Yamashita and Tanoue, 2008
Polysaccharides (laminarin, lipopolysaccharides)	Recalcitrant organic matter	Facchini et al., 2010; Cochran et al., 2017
Saccharides (glucose)	Labile organic matter	Kirchman et al., 2001; Russell et al., 2009; Frossard et al., 2014; Cochran et al., 2017; Miyazaki et al., 2018; Zeppenfeld et al., 2021
Fatty acids (palmitic acid, phospholipids, glycolipids, triacylglycerides)	Labile organic matter	Mochida et al., 2002; Cochran et al., 2017
Amphiphilic proteins (lipase)	Labile organic matter	Schiffer et al., 2018

Humic-like substances (HULIS) are a loosely defined class of large acidic or polyacidic, chromophoric and fluorescent organic molecules, which are often present in the atmosphere (Brooks et al., 2004; Dinar et al., 2007). The presence of HULIS modifies the water uptake (Brooks et al., 2004) and optical properties of aerosol (Hoffer et al., 2006) and may influence droplet formation and ice nucleation in the atmosphere (Chen et al., 2021). HULIS has been identified in marine aerosol (Cavalli et al., 2004; Krivácsy et al., 2008; Deng et al., 2014). Humic and humic-like substances in the ocean are a component of chromophoric dissolved organic matter (CDOM), which is often analyzed using optical techniques (Coble, 1996, 2007). Excitation-emission matrix spectroscopy has shown several common peaks indicating the humic components of CDOM in seawater (Coble, 1996, 2007; Stedmon and Markager, 2005). HULIS are produced by marine ecosystems (Stedmon and Markager, 2005; Murphy et al., 2008) or are transported into the ocean from terrestrial sources by rivers (Hedges et al., 1992; Huguet et al., 2009; Krachler and Krachler, 2021). The relative contributions of HULIS in marine aerosol from marine ecosystem processes, terrestrial ecosystems, and anthropogenic sources (e.g., combustion of coal and biomass; Huo et al., 2021) is uncertain. Much remains to be learned about the molecular diversity of primary marine aerosol and how it relates to processes in the underlying water. There is a need for analytical approaches that directly compare organic matter collected in both ocean and atmosphere to determine which components of marine DOM and particulate organic matter (POM) are transferred into the atmosphere and in what quantities (Brooks and Thornton, 2018).

## Emissions of Marine Microorganisms to the Atmosphere

### Virus Emissions

Viruses are the most genetically diverse and abundant (10^9^–10^12^ L^–1^; Suttle, 2005) biological entities in the ocean and play an important role in both regional and global biogeochemical cycling (Suttle, 2007; Mojica and Brussaard, 2014). Due to their high abundance and small size (20–200 nm diameter; Fuhrman, 1999), viruses are probably an important component of primary marine aerosol. Limited research shows that viruses occur in marine aerosol and are enriched in SSA up to 250-fold compared with the underlying seawater (Baylor et al., 1977; Aller et al., 2005; Leck and Bigg, 2005; Sharoni et al., 2015; Rastelli et al., 2017; Michaud et al., 2018). Viruses attach to exopolymer particles and concentrate in the SML (Aller et al., 2005). Genomic analysis of viral DNA indicates that lipid-enveloped viruses are enriched in SSA, possibly due to their hydrophobic surface properties (Michaud et al., 2018). Enveloped viruses can tolerate desiccation and withstand relative humidity (RH) as low as 15%, which is important for remaining infective during atmospheric transport (Tang, 2009).

Reche et al. (2018) found that atmospheric viruses are associated with particles, such as soil grains and marine organic aggregates. However, virus deposition was correlated with small aerosol particles (<0.7 μm diameter) compared with the larger particles (>0.7 μm diameter) associated with bacterial deposition. Based on this observation, Reche et al. (2018) hypothesized that viruses are transported further in the atmosphere than bacteria. Reche et al. (2018) measured deposition rates as two sites in the Sierra Nevada Mountains (Spain), which were located above the atmospheric boundary layer (1.7 ± 0.5 km above sea level at this location) at 2.9 and 3.0 km above sea level. Deposition rates of viruses were 9–461 times greater than those of bacteria and ranged from 0.26 × 10^9^ to >7 × 10^9^ m^–2^ per day. Back trajectories indicated higher virus deposition rates were associated with air masses originating from the Atlantic Ocean rather than terrestrial sources (Reche et al., 2018).

Viruses are a major source of microbial mortality, influencing phytoplankton bloom termination and the abundance and composition of Bacterial and Archaeal assemblages. Lytic viral infection results in the release of small POM particles and DOM composed of the major classes of organic matter (carbohydrates, proteins, lipids, and nucleic acids) (Wilhelm and Suttle, 1999; Suttle, 2005), altering the composition and distribution of organic matter, and influencing organic particle size distributions (Fuhrman, 1999). Consequently, in addition to the aerosolization of viruses themselves, interactions between viruses and their hosts have significant impact on the composition of the pool of organic matter available for aerosolization from the ocean.

### Archaea Emissions

Widely used cell counting methods, including staining cells with fluorescent probes, such as 4′,6-diamidino-2-phenylindole (DAPI) (Porter and Feig, 1980), are designed to give total counts rather than discriminate between different taxa. Consequently, many counts of microorganisms in aerosol samples do not distinguish between Bacteria and Archaea and “bacterial” counts potentially include cells from both domains of life (e.g., Aller et al., 2005; Amato et al., 2007). Archaea in the atmosphere are poorly characterized as a separate group, with a limited number of studies from urban (Radosevich et al., 2002; Brodie et al., 2007; Bowers et al., 2013; Robertson et al., 2013; Smith et al., 2013), terrestrial and coastal (Yooseph et al., 2013; Fröhlich-Nowoisky et al., 2014), and remote ocean regions (Cho and Hwang, 2011; Xia et al., 2015). The emission flux of marine Archaea into the atmosphere has not been estimated, though it is likely to be significantly less than that of Bacteria. While Archaea are numerically abundant in the ocean, they make up a small proportion (<20%) of the total prokaryote population (Karner et al., 2001; Church et al., 2003; Kirchman et al., 2007) at the ocean surface. Limited sequencing studies show that Bacteria are more prevalent than Archaea in the atmosphere (Cho and Hwang, 2011; Cáliz et al., 2018).

### Bacteria Emissions

Sea spray aerosol is enriched in bacteria compared with the underlying seawater (10–2,500-fold) (Blanchard and Syzdek, 1982; Marks et al., 2001; Aller et al., 2005; Rastelli et al., 2017). Marine air masses contain primarily Gram-negative bacteria, whereas continental air masses are dominated by Gram-positive (Fahlgren et al., 2010; Cho and Hwang, 2011; Urbano et al., 2011). This analysis suggests bacteria of marine origin are significant in air masses over the ocean as Gram-negative bacteria comprise up to 80–95% of the total bacteria counts in seawater (Watson et al., 1977). Molecular techniques, including 16S rRNA gene sequencing, quantitative PCR (qPCR) (Cho and Hwang, 2011; Urbano et al., 2011; DeLeon-Rodriguez et al., 2013; Fahlgren et al., 2015; Mayol et al., 2017; Romano et al., 2019; Uetake et al., 2020), and metagenomics (Michaud et al., 2018; Amato et al., 2019) are used to determine composition and indicate atmospheric abundances. Many non-culturable bacterial clades are emitted to the atmosphere, and therefore the community is more diverse and abundant than previously thought based on traditional culturing methods (Cho and Hwang, 2011; Cáliz et al., 2018; Uetake et al., 2020). Different regions of the ocean emit distinct bacteria to the atmosphere, resulting in significant differences in airborne microbial communities (Seifried et al., 2015; Michaud et al., 2018; Amato et al., 2019). Processes at the air-sea interface are selective and some bacteria are preferentially lofted into the atmosphere, such as mycolic acid-coated taxa with hydrophobic surface properties (Michaud et al., 2018).

It is not known whether ubiquitous marine clades, such as SAR11 (Morris et al., 2002; Giovannoni, 2017), are prevalent components of SSA. SAR11 has the potential to travel long distances in the atmosphere due to its small size (Figures 1, 2). Aerial dispersal could play a role in the population ecology and biogeography of SAR11 and other abundant clades of bacteria. Of particular interest are the cyanobacteria due to their numerical abundance in surface waters and key ecosystem role as photosynthetic primary producers. *Prochlorococcus* is the most numerically abundant photosynthetic organism on Earth (Schattenhofer et al., 2009; Flombaum et al., 2013; Biller et al., 2015), and *Synechococcus* is numerically abundant with a wider geographical distribution (Partensky et al., 1999; Flombaum et al., 2013). Sequencing of the 16S rRNA gene showed relatively low abundances of cyanobacteria in aerosol over coastal regions, though this was likely due to dilution by terrestrial heterotrophic bacteria (Cho and Hwang, 2011; Seifried et al., 2015; Xia et al., 2015; Hu et al., 2017). It is important to know if bacterial populations, particularly those separated by geographical barriers such as continents, are connected via the atmosphere sufficiently to affect their population ecology. This is a significant challenge, requiring a better understanding the population structure of bacteria in the ocean, which should be integrated into studies of bacterial population structure and viability in SSA. This approach should be applied to numerically abundant, widely distributed, and biogeochemically significant clades from the Earth’s largest biome, the open ocean.

### Eukaryote Emissions

In contrast to Bacteria and Archaea, eukaryotes are characterized by structurally complex cells that contain membrane bound structures, including a nucleus. Two groups of eukaryotes are commonly found in the marine atmosphere; protists and fungi. Protists are a diverse, and predominantly unicellular, paraphyletic group of organisms (O’Malley et al., 2013; Adl et al., 2019; Burki et al., 2020). Paraphyly refers to a grouping of organisms that are all descended from a common ancestor, but not all the descendants of the common ancestor are included in the group. For example, animals and plants share a common ancestor with all protists, but these multicellular organisms are not protists. Examples of marine protists include eukaryote phytoplankton (e.g., dinoflagellates, diatoms, coccolithophores, and chlorophytes) and heterotrophic organisms such as ciliates, foraminifera, and radiolaria (Adl et al., 2019).

The transfer of eukaryote phytoplankton to the atmosphere from the ocean occurs in the form of intact cells and recognizable cell fragments. Laboratory studies have found fragments of coccoliths from *Emiliania huxleyi* cell walls (Hawkins and Russell, 2010; Trainic et al., 2018). Diatom cells and cell fragments (including siliceous material from cell walls) were observed in several field and laboratory studies (Bigg and Leck, 2001; Leck and Bigg, 2005; Alpert et al., 2015; Lee et al., 2015; Cochran et al., 2017; Marks et al., 2019). The North Atlantic spring bloom, which is dominated by diatoms, produces organic-rich SSA (O’Dowd et al., 2004, 2015; Yoon et al., 2007; Sciare et al., 2009; Rinaldi et al., 2013). Diatoms are hypothesized to have a greater atmospheric significance than other eukaryotes due to their observed enrichment in SSA (Fuentes et al., 2010). Cell wall components could be used as biomarkers of major phytoplankton groups in marine aerosol (Table 2).

**FIGURE 5:**
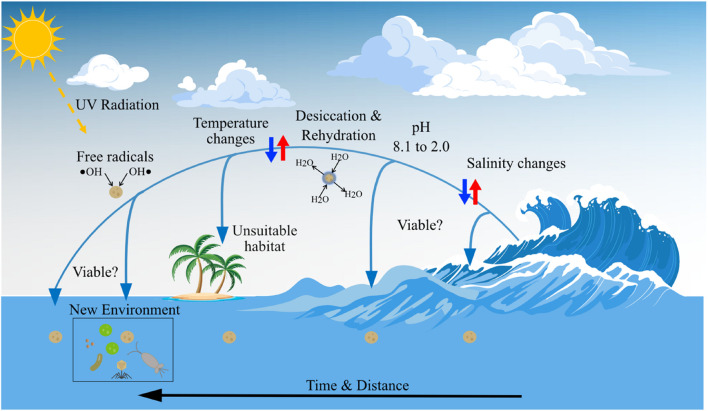
Marine microorganisms are exposed to stressors in the atmosphere that potentially reduce viability: changes in water salinity and pH, temperature change, desiccation, and rehydration, exposure to free radicals and other oxidants, exposure to solar radiation (including UV). Other stressors include the rapid rates of environmental change that can occur on transport across the air-sea interface, and deposition in an unsuitable environment. The vector art used in this figure was downloaded from vecteezy.com.

Laboratory experiments showed that rising bubbles scavenge small diatoms and eject them into the atmosphere when the bubbles burst at the air-water interface, with an enrichment factor of up to 307 in the emitted jet drops compared with the concentration in the bulk water (Marks et al., 2019). Aerial dispersal of eukaryote phytoplankton on the order of 10^2^ to 10^3^ km is possible in the troposphere (Figure 2). Airborne eukaryotes from terrestrial, coastal, and freshwater sources colonize new habitats (Schlichting, 1969; Genitsaris et al., 2011; Tesson and Santl-Temkiv, 2018) and therefore it is likely that marine eukaryote phytoplankton are also dispersed to new habitats, affecting their distribution and geographical range. The atmosphere is a relatively harsh environment for eukaryote phytoplankton (Figure 5) and little is known about how atmospheric conditions in the troposphere reduce phytoplankton viability over time. Estimates of eukaryote phytoplankton emitted to the atmosphere from remote marine regions are not known, but total eukaryote abundances in the boundary layer from the North Atlantic Ocean range from 10^2^ to 10^4^ cells m^–3^ (Mayol et al., 2014). Estimates from subtropical and tropical regions may be higher (10^2^ to 10^5^ eukaryotes m^–3^), but likely contain mostly terrestrial sources and low phytoplankton abundances (Mayol et al., 2017).

One approach to assessing biogeographic significant of eukaryotes would be to focus on widely distributed bloom forming taxa, such as diatoms and coccolithophores. The coccolithophore *Emiliania huxleyi* is globally distributed (Paasche, 2001; Taylor et al., 2017) and coccoliths from *E. huxleyi* are enriched in SSA (Trainic et al., 2018). The optical properties of *E. huxleyi* mean that regional blooms, with length scales of 10^3^ kilometers (Balch, 2018) are quantifiable from satellite observations. It would be possible to track the composition and properties of air-masses over a significant period of time as they pass over a bloom. Given the long length and timescales of *E. huxleyi* blooms, it may be possible to link composition of atmosphere and ocean during these events. In the laboratory, controlled experiments using a marine aerosol reference tank (MART) (Fuentes et al., 2010; Stokes et al., 2013) could be used to investigate the potential aerosolization of specific phytoplankton taxa with ecological and biogeochemical significance. For example, ubiquitous picoeukaryotes (<2 μm diameter) (de Vargas et al., 2015), such as *Micromonas* sp. and *Ostreococcus* sp., have the potential to be transported great distances in the atmosphere (Figure 2).

Fungi and heterotrophic protists have been observed in a limited number of studies over the open ocean (Mayol et al., 2017) and in coastal regions (Marks et al., 2001; Prospero et al., 2005; Fu et al., 2013; Genitsaris et al., 2014; Cáliz et al., 2018). The majority of fungal biomass in the ocean consists of single-celled Ascomycota species (Shearer et al., 2007). Their smaller size (less than 3 μm) indicates they may be transported longer distances than the primarily terrestrial fungal group, the Basidiomycota (greater than 3 μm) (Fröhlich-Nowoisky et al., 2009, 2012). On a global scale, marine fungal spore emissions are several orders of magnitude smaller than terrestrial emissions (Elbert et al., 2007; Heald and Spracklen, 2009; Fröhlich-Nowoisky et al., 2012). Using 18S rRNA gene sequencing, Cáliz et al. (2018) determined >75% of total eukaryotes from the atmosphere belonged to Ascomycota and Basidiomycota at a coastal site in the Mediterranean, with air masses originating from both open ocean and continental sources. Fungal spores can survive harsh environmental conditions during atmospheric dispersal (Fröhlich-Nowoisky et al., 2016) and may have relatively long viable residence times. It is not clear how far fungi and other eukaryotes travel in the atmosphere. Potentially, a cell 3 μm in diameter could be dispersed over 30,000 km, starting at an altitude of 1,000 m with a wind speed of 10 m s^–1^ (see Figure 2 for an explanation). This journey would take 35 days; it is unlikely that viable eukaryotes travel such distances as they would be returned to the ocean through wet deposition en route, or lose viability in the atmosphere.

## Ecological Significance of Marine Microorganisms in the Atmosphere

Biogeographical patterns of microorganisms in the ocean are mainly determined by local selection and dispersal mechanisms (Hanson et al., 2012), such as relatively slow-moving oceanic currents and fast-moving winds (Wilkins et al., 2013). Community similarity decreases with increasing geographical distance due to limitations of dispersal and adaptation (Morlon et al., 2008; Nemergut et al., 2013). Relatively few studies have focused on the ecological implications of airborne microbial communities (Kellogg and Griffin, 2006; Smith et al., 2013). Potentially, the dispersal of viable marine microorganisms through the atmosphere would enable them to overcome environmental barriers (Figure 5) between suitable habitats (Hamilton and Lenton, 1998; Thornton, 1999). Such aerial connectivity may contribute to the genetic composition (Sharma and Singh, 2010; Womack et al., 2010) and emergent phenotypic traits of populations. For example, *Prochlorococcus* is known to have distinct ecotypes found in different environments, with differences in both physiology and geographical distribution (Rocap et al., 2003; Biller et al., 2015). Tracking the potential aerial emission and dispersal of different *Prochlorococcus* genotypes using molecular techniques could provide insight into genetic dispersal, variability between distant marine regions, and the evolution of the different ecotypes observed today.

After emission, marine microorganisms may travel for several days, with an estimated 10% remaining in the atmosphere after 4 days (Mayol et al., 2014), covering distances more than sufficient to traverse the Atlantic or Indian Oceans. The deposition of marine microorganisms was estimated to be 9.85 eukaryotes m^–2^ s^–1^ and 49.00 prokaryotes m^–2^ s^–1^ over the North Atlantic Ocean, indicating it would take up to 0.7 days to deposit 50% of aerosolized microorganisms (Mayol et al., 2014). It is difficult to extrapolate these estimates to the global ocean because so few studies have been conducted to determine fluxes and deposition rates (Burrows et al., 2009a). Larger eukaryotic phytoplankton have residence times of less than 1 day and potential to travel a few hundred kilometers, whereas smaller microorganisms (<5 μm), such as cyanobacteria, SAR11, and viruses, have potential residence times of years and travel much longer distances (Figure 2). Calculations of atmospheric residence time and distance transported based on organism size are likely to be overestimates as most atmospheric microorganisms are associated with larger particles (Reche et al., 2018). Atmospheric residence time is based on the gravitational forces associated with mass, as well as the drag forces associated with size, density, and shape (Tesson et al., 2016). Additionally, atmospheric properties such as wind speed, direction, and precipitation affect distance traveled. Atmospheric structures, such as clouds and fog, may limit dispersal to higher altitudes (Carson and Brown, 1976). Microorganisms are deposited by dry or wet deposition (Tesson et al., 2016). Dry deposition includes settling and impaction of particles under the influence of meteorological conditions such as wind, RH, and temperature (Tesson et al., 2016). Wet deposition is a major removal mechanism of microorganisms from the atmosphere (Burrows et al., 2009b), which limits exposure to environmental stressors by significantly reducing atmospheric residence time (Tesson et al., 2016).

It is estimated that the minority (1–25%) of microorganisms emitted to the atmosphere are viable upon deposition (Womack et al., 2010; Polymenakou, 2012). If aerosolization and processing in the atmosphere results in cell death, then aerial transport cannot play a significant role in determining ecosystem structure, population genetics, or colonization of distant locations. During atmospheric dispersal marine microorganisms face harsh environmental conditions (Figure 5), including increased UV radiation exposure, desiccation and rehydration, temperature changes, exposure to free radicals and other oxidants, rapid salinity changes, relatively low pH, and limited nutrients for growth (Marthi et al., 1990; Walter et al., 1990; Womack et al., 2010; Tesson et al., 2016; Tesson and Santl-Temkiv, 2018; Wilbourn et al., 2020; Angle et al., 2021). Studies of aerosolization have indicated viability is dependent on temperature, RH, salt concentration, and droplet size (Marthi et al., 1990; Walter et al., 1990). Alsved et al. (2018) concluded, from experiments with the terrestrial plant bacterial pathogen *Pseudomonas syringae*, that the conditions under which cells dry in the atmosphere are key to determining viability. Marine microorganisms in SSA are likely to be exposed to significant and rapid changes in salinity during atmospheric transport and therefore significant osmotic challenges. Freshly emitted SSA has the salinity of local seawater, which is significantly reduced during cloud formation processes to due to rapid uptake of water by aerosol particles (Wilbourn et al., 2020). Conversely, salt within SSA may increase to concentrations several times that of seawater as water evaporates from an aerosol particle. The pH of the ocean is 8.1; airborne microorganisms are exposed to acidic conditions (pH ∼2) in fresh SSA (Angle et al., 2021) and precipitation (pH ∼5.6) (Charlson and Rodhe, 1982). Exposure to UV radiation reduces viability, but many microorganisms produce pigments (e.g., carotenoids) or contain high DNA guanine-cytosine (GC) content (Matallana-Surget et al., 2008) to avoid photodamage.

Culturing organisms isolated from aerosol samples is a proven method for determining viability (Amato et al., 2005; 2007; Ahern et al., 2007; Renard et al., 2016), though only a minority (<1%) of prokaryotes in the ocean and atmosphere are amenable to culture using current methods (Amato et al., 2005, 2007; Kirchman et al., 2009; Fröhlich-Nowoisky et al., 2016). The vast majority of taxa (>99%) are simply ignored in abundance and community composition studies dependent on culturing methods. Studies combining culture-independent (e.g., 16S and 18S RNA gene sequencing) and culture-dependent approaches illustrate the limitations of culturing, with sequencing methods showing higher abundances and a more diverse population (Cho and Hwang, 2011; Ravva et al., 2012; Smith et al., 2012; DeLeon-Rodriguez et al., 2013). Culture-independent methods indicate the presence of a particular operational taxonomic unit (OTU), but not whether it was alive in the aerosol. Culture-dependent methods directly show viability, but only for the limited number of taxa that are selected for by the culture conditions. A lack of standard sampling methods makes interpretation and comparison between studies challenging (Burrows et al., 2009a).

Methods are needed that combine the power and resolution of culture-independent methods with measurements of viability. Viability is commonly determined using live/dead staining techniques based on fluorescent probes (Hu et al., 2017, 2020; Alsved et al., 2018). These methods provide information on the relative proportion of viable cells in a sample, but not which taxa are viable. New molecular based methods, such as viability PCR (vPCR), could be used to not only characterize the atmospheric microbial community but also differentiate viable from non-viable cells (Cangelosi and Meschke, 2014; Baymiev et al., 2020). Live/dead staining and vPCR are not direct measures of viability; it is assumed that cell permeability indicates compromised cell membranes and therefore non-viable cells. Active gene expression provides an alternative approach to assay for viability in airborne microbial communities, using methods such as serial analysis of gene expression (SAGE) (Velculescu et al., 1995; Hu and Polyak, 2006). Even defining living and dead microorganisms is complex and debated (Emerson et al., 2017), which raises the question of whether using a single method is conclusive. The current list of available culture-dependent and independent studies of airborne microbial communities is summarized in Supplementary Table 3.

A major unknown is what proportion of the marine aerobiota is metabolically active and whether this has a significant effect on aerosol-cloud interactions and the processing of marine DOM in the atmosphere. Cloud water contains DOM, which could support microbial metabolism (Khaled et al., 2020), including the remineralization of DOM back to carbon dioxide. Ervens and Amato (2020) estimated that the global loss of DOM in clouds is 0.008–0.011 Pg C yr^–1^, which is insignificant when compared with the 662 Pg C as DOM in the ocean (Hansell et al., 2009), or estimates of annual oceanic photosynthetic production of 45–55 Pg C yr^–1^ (Longhurst et al., 1995; Field et al., 1998; Carr et al., 2006; Westberry et al., 2008). It seems highly unlikely that overlooking the microbial remineralization of organic matter in the atmosphere has resulted in significant error in global carbon cycling models. Untargeted metatranscriptomics of samples collected in clouds at a terrestrial site (Puy de Dôme mountain, France) indicated a diverse range of metabolic processes within active cells; including energy metabolism, stress responses, transcription and translation, transport, and biosynthesis (Lallement et al., 2018; Amato et al., 2019). To date, metatranscriptomic approaches have not been used to determine microorganism activity in SSA collected over the ocean. Cell division within aerosol and rainwater has been shown (Dimmick et al., 1979; Herlihy et al., 1987; Sattler et al., 2001). Ervens and Amato (2020) estimated that bacterial growth and cell division generates 3.7 Tg yr^–1^ of secondary biological aerosol globally. This estimate is poorly constrained due to limited data. Research on living microorganisms in the atmosphere is in its infancy, and the connections between marine microorganisms and microbial activity in the atmosphere are unknown. At present, it is not known whether metabolically active bacteria are rare exceptions or whether there are microbial communities forming the network of interactions associated with ecosystems (Smets et al., 2016).

The preceding discussion has focused on cellular organisms, but the aerial dispersal of viable viruses has implications for their host populations, in addition to the biogeography and population genetics of the viruses themselves. *Emiliania huxleyi* viruses (*Eh*Vs) affect regional-scale biogeochemical processes due to their role in terminating *Emiliania huxleyi* blooms (Bratbak et al., 1993; Lehahn et al., 2014; Sharoni et al., 2015). *Emiliania huxleyi* viruses (*Eh*Vs) are emitted as primary aerosol, with evidence that they are transported and remain infective over hundreds of kilometers (Sharoni et al., 2015).

In conclusion, aerial dispersal is an overlooked, but potentially important, mechanism that may lead to a better understanding of marine microbial biogeography. Atmospheric dispersal has important implications for microbial assemblage composition and genetic diversity through ecological processes such as horizontal gene transfer and competition (Womack et al., 2010; Nemergut et al., 2013). However, more data are needed on biogenic aerosol emission, transport, and particularly viability, before we can integrate atmospheric processes into our models of microbial oceanography, and biogeochemistry.

## Effect of Aerosolized Marine Organic Matter on Atmospheric Processes

Aerosol plays a significant role in climate by directly scattering or absorbing solar radiation and indirectly by affecting cloud properties by acting as INPs or CCN (Boucher et al., 2013; Figure 4). SSA is the dominant driver of light scattering in the marine boundary layer (Lu et al., 2015; Quinn et al., 2015) and global modeling suggests SSA radiative forcing could be greater than natural continental sources, such as mineral dust or sulfate aerosol particles (Jacobson, 2001; Takemura et al., 2002). A fraction of the organic matter in SSA will be chemically altered in the atmosphere due to solar radiation exposure, acidic conditions, and oxidation (Mopper et al., 1991; Keene et al., 2007; Frossard et al., 2014; Kieber et al., 2016; Beaupré et al., 2019; Figure 5). Chemical degradation forms inorganic volatile carbon species (e.g., CO_2_ and CO; Kieber et al., 2016; Beaupré et al., 2019) or low-molecular-weight organic compounds such as aldehydes, ketones, and organic acids (Frossard et al., 2014; Kieber et al., 2016), and inorganic species containing nutrients (N and P) (Beaupré et al., 2019). The photochemical oxidation of marine recalcitrant organic matter in the atmosphere affects the formation of inorganic carbon, or labile molecules that are remineralized to inorganic carbon by heterotrophic bacteria on deposition in the ocean. Thus, atmospheric photochemical oxidation is a poorly constrained sink for marine recalcitrant organic matter (Keene et al., 2015; Kieber et al., 2016). Processing of organic matter in SSA that alters fundamental properties, such as particle size and chemical composition, modify the ability of aerosol to act as CCN and INPs.

Some studies have shown that primary marine aerosol enriched in organic matter has increased CCN activity (Meskhidze et al., 2011; Ovadnevaite et al., 2011), while other work showed that organic matter reduces CCN activity (Fuentes et al., 2011) or has little to no effect on CCN activation (Deng et al., 2014; Schwier et al., 2015; Collins et al., 2016; Bates et al., 2020; Hendrickson et al., 2021). Sea salt is a major source of CCN in remote marine regions (O’Dowd et al., 1997; Clarke et al., 2006). Due to the large emission of sea salt to the atmosphere (2,000–10,000 Tg yr^–1^) and small contribution of organic matter (10 ± 5 Tg yr^–1^) (Gantt and Meskhidze, 2013), organics likely have a small or negligible effect on CCN activity, especially in SSA particles that are a mixture of sea salt and organic matter, as there is simply not enough organic matter to affect CCN properties (Hendrickson et al., 2021) (see Supplementary Table 1). In addition, it is challenging to establish links between ecosystems and CCN due to the complexity and different timescales of processes in both ocean and atmosphere. For example, coccolithophore blooms are proposed as a major source of DMS (Malin et al., 1993; Matrai and Keller, 1993), which is oxidized in the atmosphere to form aerosol that activate as CCN (Charlson et al., 1987; Quinn and Bates, 2011). Laboratory experiments showed that viral infection induces the coccolithophore, *Emiliania huxleyi*, to shed coccoliths, which became enriched in SSA (Trainic et al., 2018). Coccolith enrichment in SSA potentially increases cloud droplet alkalinity, leading to reactions between sulfur dioxide and ozone, and reducing CCN activity from DMS-derived sulfur (Sievering et al., 2004; Trainic et al., 2018).

Clouds containing ice are present at all latitudes (Shupe et al., 2008; Korolev et al., 2017), affecting Earth’s radiative budget and precipitation patterns (Boucher et al., 2013; Vali et al., 2015). Homogeneous freezing of pure water droplets in the atmosphere occurs below −38°C due to the stearic challenge of freezing in tiny droplets (Hoose and Möhler, 2012; Kanji et al., 2017). Heterogeneous freezing catalyzed by INPs occurs at warmer temperatures, although still below 0°C (Vali, 1971; Hoose and Möhler, 2012). Heterogeneous freezing drives the formation and properties of mixed-phase and ice clouds in the troposphere. Although the composition of an effective INP remains poorly understood, there are certain characteristics that promote ice nucleation. These include a crystalline structure, large particle surface area, an amorphous semi-solid or viscous liquid phase (Hoose and Möhler, 2012; Collier and Brooks, 2016; Kanji et al., 2017; Knopf et al., 2018). Efficient INPs may contain hydroxyl and amino groups that initiate ice formation via hydrogen bonding to water molecules (Kanji et al., 2017). Sea salt suppresses the freezing temperature of INPs and therefore inhibits the ability of organic-rich aerosol to efficiently act as an INP (Wilbourn et al., 2020). However, the activation of SSA as CCN dilutes the salt enabling marine organic matter to act as efficient immersion INPs (Wilbourn et al., 2020). Global modeling indicates marine organics may be an important source of INPs, particularly in remote marine regions (Burrows et al., 2013; McCluskey et al., 2019).

Both laboratory and field studies indicate SSA generated from biologically productive marine waters nucleate ice moderately efficiently (i.e., 5–15°C warmer than homogeneous nucleation) (Schnell and Vali, 1976; DeMott et al., 2016; Wilbourn et al., 2020). Ice nucleation of biogenic aerosol may change depending on phytoplankton physiological status (Prather et al., 2013; McCluskey et al., 2017), and phytoplankton senescence is proposed as a major source of INPs due to the release of organic matter (McCluskey et al., 2018). INPs are associated with a diverse range of phytoplankton groups, including diatoms (Knopf et al., 2010; Alpert et al., 2011a; Wilson et al., 2015), coccolithophores (Alpert et al., 2011b), cyanobacteria (Wolf et al., 2019; Wilbourn et al., 2020), and chlorophytes (Alpert et al., 2011b). Wilbourn et al. (2020) used flow cytometry to sort phytoplankton from the North Atlantic by size and determined picoeukaryotes (1–3 μm), nano-eukaryotes (3–50 μm), and the cyanobacterium, *Synechococcus* (0.5–1 μm), were all moderately efficient at ice nucleation in the immersion mode. There is a significant amount of data showing that marine biogenic INPs consistently freeze ∼10°C warmer than homogeneous freezing in the immersion mode, which suggests a common, though unknown, property affecting ice nucleation.

Several studies have shown INPs are present in seawater samples that have been filtered and contain particles <0.3 μm in diameter (Rosinski et al., 1987; Wilson et al., 2015; DeMott et al., 2016; Irish et al., 2017). These results indicate that organic INPs are not whole cells, which are generally >0.5 μm in length (Ducklow, 2001). Size and chemical composition suggests that viruses may be important in the formation of ice in clouds. Marine viruses are within the size class (<0.3 μm in diameter) of known INPs (Rosinski et al., 1987; Wilson et al., 2015; DeMott et al., 2016; Irish et al., 2017) and viral capsids are composed of protein (Jover et al., 2014) and therefore contain hydroxyl and amino groups. However, the only known study of marine viruses concluded that they are not efficient INPs (Junge and Swanson, 2008). In contrast, recent measurements determined that in some locations, supermicron aerosol particles may contain a significant proportion of the total INP population, including those freezing at the warmest temperatures (Mason et al., 2016; Creamean et al., 2018; Si et al., 2018; Gong et al., 2020; Mitts et al., 2021). Collectively, these results show that marine INPs are a range of sizes and that both cell fragments and whole cells are potential sources of biogenic INPs.

## Conclusion and Future Research

Ocean aerobiology offers an interdisciplinary framework that integrates microbial oceanography, biogeochemistry, atmospheric sciences, climate science, and biogeography to understand the significance of marine organic matter in the atmosphere both today and in response to future climate change. Ocean aerobiology has been overlooked compared with research on microorganisms in ocean water, despite the daily exchange of hundreds of trillions of microorganisms between ocean and atmosphere (Mayol et al., 2014, 2017). Biogeochemical processes in the ocean and overlying atmosphere are often regarded as separate, which downplays the significance of processes occurring across the air-sea interface, and the connection between physical and chemical processes in the atmosphere with biology in the ocean (Brooks and Thornton, 2018).

The potential for marine microorganisms to be dispersed thousands of kilometers in the troposphere within days indicates atmospheric transport could provide a mechanism for dispersal between ocean basins and population connectivity. Under sampling means we know relatively little about which taxa of marine microorganism are found in the atmosphere, their distribution, variability, and seasonality. Characterization of marine aerobiota in space and time is insufficient to address fundamental ecological questions; characterization of community composition must be coupled with an understanding of where organisms originated and where they are deposited, emphasizing a need for aerosol transport modeling applied to ocean aerobiology. Viability is key to determining whether intact marine microorganisms functionally and genetically link distant marine ecosystems via the atmosphere, or are merely a source of organic matter in SSA. Gene expression and viability assays should be employed to determine the potential for airborne marine microorganisms to colonize and grow in new environments, including in the atmosphere itself. A list of stressors that impact marine microorganisms in the atmosphere have been identified (Figure 5), but the physiology of acclimation to those stressors is poorly understood, as well as how different stressors interact. It is likely that the most rapid and greatest magnitude changes in environmental conditions occur during the initial (i.e., aerosolization) and final (i.e., deposition) steps of the atmospheric journey, which may be key in determining controls on viability. The physical and chemical microhabitat of SSA has not been characterized and may play a role moderating microorganisms’ exposure to atmospheric stressors (e.g., UV radiation).

The organic composition of SSA is poorly characterized and there is still much to be learned about how processes, such as bubble bursting, determine which microorganisms and components of DOM are enriched in sea spray. Linking organic matter composition in the atmosphere and ocean requires sampling procedures and analytical techniques conducive for direct and quantitative comparison of organic matter in both (Brooks and Thornton, 2018). This chemical complexity presents analytical and conceptual challenges. Targeted analyses and biomarkers (Table 2) for specific taxa or chemical pathways provide a route to simplify complexity and test specific hypotheses. Biomarkers are limited in that their application requires prior knowledge of the system. While untargeted analyses using ultra-high- resolution mass spectrometry and multivariate statistics (Kujawinski et al., 2009; Tolić et al., 2017) overcomes this limitation, interpreting the large volume of complex chemical data generated by these approaches is challenging.

Marine organic matter is an important source of INPs and may significantly contribute to the formation and properties of mixed-phase and ice clouds on a global scale (DeMott et al., 2016; Brooks and Thornton, 2018). Further work is needed to understand which components of the organic matter in SSA act as INPs. Once structures or compounds that are effective INPs have been identified, then it may be possible to address the fundamental microphysical question of what makes an effective organic INP. Furthermore, identifying the source of INPs will make it possible to design experiments to determine the physiological or ecosystem processes in the water column that produce INPs. A long-term goal should be to construct predictive models that connect marine ecosystems to SSA and their effects on cloud microphysics and climate. As much of the organic matter in marine primary aerosol is derived from phytoplankton, models based on phytoplankton growth and distribution offer the most potential for linking ecosystem processes to the chemical composition of SSA and its atmospheric properties. For example, phytoplankton resource allocation models (Droop, 1983; Geider and La Roche, 2002; Klausmeier et al., 2004) describe phytoplankton growth and chemical composition based on resource availability and physiology. Such models could be used to predict the composition of organic matter produced by phytoplankton based on environmental conditions. The global distribution of different functional groups of phytoplankton in the ocean can be predicted using trait-based models (Follows et al., 2007; Litchman and Klausmeier, 2008; Follows and Dutkiewicz, 2011).

Bioaerosols are ubiquitous in the atmosphere, but are one of the least understood components of the Earth’s biosphere (Fröhlich-Nowoisky et al., 2016; Amato et al., 2017, 2019). Bioaerosols have direct societal implications due to their role in human health, agriculture, and climate (Fröhlich-Nowoisky et al., 2016). The constant exchange of marine microorganisms with the atmosphere shows that the ocean surface is not a hard boundary to marine ecosystems. There is a need to mechanistically understand and quantify the impact of marine organisms and organic matter in the atmosphere. Ocean aerobiology has implications for ecosystem structure and function, biogeochemical cycles, weather, climate, and human wellbeing.

## Author Contributions

DT and AA conceived and planned the review. AA wrote the first draft of the manuscript, compiled the tables, and made the figures. DT and SB, in consultation with AA, revised and edited the manuscript prior to submission. All authors contributed to the article and approved the submitted version.

## Conflict of Interest

The authors declare that the research was conducted in the absence of any commercial or financial relationships that could be construed as a potential conflict of interest.

## Publisher’s Note

All claims expressed in this article are solely those of the authors and do not necessarily represent those of their affiliated organizations, or those of the publisher, the editors and the reviewers. Any product that may be evaluated in this article, or claim that may be made by its manufacturer, is not guaranteed or endorsed by the publisher.

## Abbreviations

CCN: cloud condensation nuclei
CSP: Coomassie staining particles
DMS: dimethyl sulfide
DOM: dissolved organic matter
INPs: ice nucleating particles
MART: marine aerosol reference tank
OTU: operational taxonomic unit
POM: particulate organic matter
RH: relative humidity
SAGE: serial analysis of gene expression
SML: sea surface microlayer
SSA: sea spray aerosol
TEP: transparent exopolymer particles
VOCs: volatile organic compounds
vPCR: viability polymerase chain reaction.
