# Supplementary material for: Ocean Aerobiology
Source: Front Microbiol. 2021 Oct 29;12:764178. doi: 10.3389/fmicb.2021.764178 (PMC8586456; doi:10.3389/fmicb.2021.764178)
Supplement: Supplementary file 1 [file Data_Sheet_1.docx]

Supplementary Material

# Supplementary Tables

| **Organic carbon** | **Sea salt** | **Reference** |
| --- | --- | --- |
| 14 | Unspecified | (Duce, 1978) |
| 6.3 (sub-micron) | Unspecified | (Gantt et al., 2012) |
| 3 | Unspecified | (Jaeglé et al., 2011) |
| 5.8 | 6297 | (Vignati et al., 2010) |
| 8 (primary and secondary sources)  5.5 (sub-micron WIOC) | Unspecified | (Spracklen et al., 2008) |
| 2.3-2.8 (WIOC) | Unspecified | (Langmann et al., 2008) |
| 13 (sub-micron) | Unspecified | (Lapina et al., 2011) |
| 75 | Unspecified | (Roelofs, 2008) |
| 2.9 (sub-micron WIOC)  19.4 (super-micron WIOC) | Unspecified | (Gantt et al., 2009) |
| 29 | 1500 | (Long et al., 2011) |
| 7.9-9.4 (sub-micron)  8.6-23 (super-micron) | Unspecified | (Meskhidze et al., 2011) |
| 17.7 | Unspecified | (Westervelt et al., 2012) |
| Unspecified | 5900 | (Gong, 2003) |

Table S1: Total estimated global emission rates (Tg yr^-1^) of sea salt and organic carbon in marine aerosol

* All values refer to total organic carbon unless noted as water-insoluble organic carbon (WIOC).

Table S2: Measured concentrations (µg m^-3^) of organic carbon and sea salt in marine aerosol

| **Location** | **Biological activity** | **Organic carbon** | **Sea salt** | **Reference** |
| --- | --- | --- | --- | --- |
| Northeast Atlantic (Mace Head) | HBA | 0.619 | Unspecified | (O’Dowd et al., 2004) |
|  | LBA | 0.07 |  |  |
| Northeast Atlantic (Mace Head) | HBA | 0.36-3.8 | Unspecified | (Ovadnevaite et al., 2011) |
| Northeast Atlantic (Mace Head) | HBA | 0.34-0.44 (WIOC) | 0.52-0.55 | (O’Dowd et al., 2008) |
|  | LBA | 0.1 (WIOC) | 1.51 |  |
| Northeast Atlantic (Mace Head) | HBA | 1.2; 0.2 (WSOC) | Unspecified | (Yoon et al., 2007) |
|  | LBA | 0.1; 0.05 (WSOC) |  |  |
| Northeast Atlantic (Mace Head) | HBA | 0.11 | Unspecified | (Mansour et al., 2020) |
| Northwest Atlantic (Sargasso Sea) | Unspecified | 1.45-1.75 | Unspecified | (Keene et al., 2007) |
| Southeast Pacific | LBA | 0.01 | Unspecified | (Shank et al., 2012) |
| Northern Indian Ocean | Unspecified | 0.06-1.7 | Unspecified | ﻿(Neusüß et al., 2002) |
| Southern Indian Ocean | HBA | Maximum of >0.2 | Unspecified | (Sciare et al., 2009) |
|  | LBA | Minimum of <0.1 |  |  |
| Arctic | HBA | 0.11-2.93 | Unspecified | (Fu et al., 2013) |
| Arctic and North Atlantic | Unspecified | 0.1-0.4 | Unspecified | (Russell et al., 2010) |

* All values refer to total organic carbon unless noted as water-insoluble organic carbon (WIOC) or water-soluble organic carbon (WSOC). Organic carbon values correspond to high biological activity (HBA), or low biological activity (LBA) determined by chlorophyll-*a* concentration.

Table S3: Overview of studies with observations of marine microorganisms in the atmosphere

| **Location** | **Sampling Method** | **Culture-independent techniques** | **Culture-dependent techniques** | **Observed microorganisms** | **Citation** |
| --- | --- | --- | --- | --- | --- |
| North Atlantic Ocean | Cyclonic collector | Epifluorescence microscopy | None used | Bacteria, Protists, Unicellular Eukaryotes | (Mayol et al., 2014) |
| North Atlantic Ocean | PM10 air sampler | Targeted PCR, TEM, qPCR | Culturing (Liquid medium) | Eukaryotes (*Emiliania huxleyi*), Viruses (*Eh*V) | (Sharoni et al., 2015) |
| Northeast Atlantic Ocean (Mace Head) | Passive deposition sampler | 16S and 18S rRNA gene sequencing | None used | Bacteria, Archaea, Protists, Fungi | (Cáliz et al., 2018) |
| Atlantic Ocean | PM10 air sampler | 16S rRNA gene sequencing, qPCR | None used | Bacteria, Archaea | (Fröhlich-Nowoisky et al., 2014) |
| Carribean Sea, Gulf of Mexico, and Midwestern Atlantic Ocean | Vacuum pump | Epifluorescence microscopy, 16S rRNA gene sequencing, and qPCR | None used | Bacteria, Fungi | (DeLeon-Rodriguez et al., 2013) |
| Atlantic and Arctic Ocean | Impinger | 16S rRNA gene sequencing | Culturing (Agar plate and Mesocosms) | Bacteria, Phytoplankton | (Fahlgren et al., 2015) |
| Northern and Western Pacific Ocean, Actic Ocean, and Norwegian Sea | Cascade impactor | 16S rRNA gene sequencing | None used | Bacteria | (Xia et al., 2015) |
| Pacific Ocean | Spore trap | 16S and 18S rRNA gene sequencing | None used | Phytoplankton | (Sherwood et al., 2020) |
| Pacific Ocean | Cascade impactor | 16S and 18S rRNA gene sequencing | Culturing (Agar plate) | Fungi, Bacteria | (Urbano et al., 2011) |
| Atlantic, Pacific, and Indian Ocean | Cyclonic collector | Epifluorescence microscopy, 16S rRNA gene sequencing | None used | Bacteria, Archaea, Fungal spores, Protists | (Mayol et al., 2017) |
| East and South China Sea, South Pacific, East Indian, South Atlantic, and Southern Ocean | High-volume filter sampler | 5S rRNA gene sequencing | None used | Fungi | (Fröhlich-Nowoisky et al., 2012) |
| South Baltic Sea | Cascade impactor | Light and epifluorescence microscopy | Culturing (Liquid medium) | Phytoplankton | (Lewandowska et al., 2017) |
| Baltic Sea | Sartorius air filtration unit | None used | Culturing (Agar plate) | Bacteria | (Marks et al., 2001) |
| North Sea and Baltic Sea | Impinger | 16S rRNA gene sequencing | None used | Bacteria | (Seifried et al., 2015) |
| East Sea | Impinger and Filtration | Epifluorescence microscopy, 16S rRNA gene sequencing, and qPCR | Culturing (Agar plate) | Bacteria, Archaea | (Cho and Hwang, 2011) |
| Southeast Mediterranean Sea | High-volume filter sampler | Flow cytometry, 16S rRNA gene sequencing, and Bacterial enzyme activity | Culturing (Microcosms) | Bacteria | (Rahav et al., 2016) |
| Southern Ocean | Filtration | 16S rRNA gene sequencing | None used | Bacteria, Archaea | (Uetake et al., 2020) |
| Arctic Ocean | Impactor and Electrostatic precipitator | TEM | None used | Bacteria, Viruses | (Leck and Bigg, 2005) |
| Arctic Ocean | Impactor and Electrostatic precipitator | TEM | None used | Bacteria, Diatoms | (Bigg and Leck, 2001) |

*TEM refers to transmission electron microscopy

**Figures 1 and 2 calculations: deposition velocity, distance travelled, and residence time**

The deposition velocity for an aerosol particle was calculated using Stokes’ Law where $p_{p}$ is the particle density $\left( g/{cm}^{3} \right)$, $d_{p}$ is the diameter of the particle$\left( cm \right)$, $g$ is the acceleration of gravity $(9.8\times{10}^{2}cm/s)$, $\mu$ is the coefficient of air viscosity ($g/cm s)$, and $\lambda$ is the mean free path of gas molecules $\left( cm \right).$ The Cunningham Correction Factor was used for particles with a diameter of < 1 $\mu$m to account for slippage past gas molecules. Settling velocities were calculated at the average tropospheric temperature (15°C). Starting altitudes (0 m, 250 m, 500 m, 750 m, and 1000 m) assumed the particle would be lofted into the atmosphere at this height and the distance travelled and residence time in the atmosphere was calculated using an average windspeed of 10 m s^-1^. An average density of 1.1 $\left( g/{cm}^{3} \right)$ was used for biological particles (Mayol et al., 2014), and a relative humidity of 70% was used to determine density and viscosity in the troposphere (Williams, 1982).

$$Equation 1 \left( Stokes' Law \right): V_{t}= \frac{p_{p}d_{p}^{2}gC^{c}}{18\mu}$$

$${Equation 2 \left( Cunningham Correction Factor \right): C}^{c}=1+\frac{2.52\lambda}{d_{p}}$$

**References**

Bigg, E. K., and Leck, C. (2001). Properties of the aerosol over the central Arctic Ocean. *J. Geophys. Res. Atmos.* 106, 32101–32109. doi: 10.1029/1999JD901136.

Cáliz, J., Triadó-Margarit, X., Camarero, L., and Casamayor, E. O. (2018). A long-term survey unveils strong seasonal patterns in the airborne microbiome coupled to general and regional atmospheric circulations. *Proc. Natl. Acad. Sci. USA* 115, 12229–12234. doi: 10.1073/pnas.1812826115.

Cho, B. C., and Hwang, C. Y. (2011). Prokaryotic abundance and 16S rRNA gene sequences detected in marine aerosols on the East Sea (Korea). *FEMS Microbiol. Ecol.* 76, 327–341. doi: 10.1111/j.1574-6941.2011.01053.x.

DeLeon-Rodriguez, N., Lathem, T. L., Rodriguez, L. M., Barazesh, J. M., Anderson, B. E., Beyersdorf, A. J., et al. (2013). Microbiome of the upper troposphere: species composition and prevalence, effects of tropical storms, and atmospheric implications. *Proc. Natl. Acad. Sci. USA* 110, 2575–2580. doi: 10.1073/pnas.1212089110.

Duce, R. A. (1978). Speculations on the budget of particulate and vapor phase non-methane organic carbon in the global troposphere. *Pure Appl. Geophys.* 116, 244–273. doi: 10.1007/BF01636883.

Fahlgren, C., Gómez-Consarnau, L., Zábori, J., Lindh, M. V., Krejci, R., Mårtensson, E. M., et al. (2015). Seawater mesocosm experiments in the Arctic uncover differential transfer of marine bacteria to aerosols. *Environ. Microbiol. Rep.* 7, 460–470. doi: 10.1111/1758-2229.12273.

Fröhlich-Nowoisky, J., Burrows, S. M., Xie, Z., Engling, G., Solomon, P. A., Fraser, M. P., et al. (2012). Biogeography in the air: Fungal diversity over land and oceans. *Biogeosciences* 9, 1125–1136. doi: 10.5194/bg-9-1125-2012.

Fröhlich-Nowoisky, J., Ruzene Nespoli, C., Pickersgil, D. A., Galand, P. E., Müller-Germann, I., Nunes, T., et al. (2014). Diversity and seasonal dynamics of airborne archaea. *Biogeosciences* 11, 6067–6079. doi: 10.5194/bg-11-6067-2014.

Fu, P. Q., Kawamura, K., Chen, J., Charrière, B., and Sempéré, R. (2013). Organic molecular composition of marine aerosols over the Arctic Ocean in summer: Contributions of primary emission and secondary aerosol formation. *Biogeosciences* 10, 653–667. doi: 10.5194/bg-10-653-2013.

Gantt, B., Johnson, M. S., Meskhidze, N., Sciare, J., Ovadnevaite, J., Ceburnis, D., et al. (2012). Model evaluation of marine primary organic aerosol emission schemes. *Atmos. Chem. Phys.* 12, 8553–8566. doi: 10.5194/acp-12-8553-2012.

Gantt, B., Meskhidze, N., and Kamykowski, D. (2009). A new physically-based quantification of marine isoprene and primary organic aerosol emissions. *Atmos. Chem. Phys.* 9, 4915–4927. doi: 10.5194/acp-9-4915-2009.

Gong, S. L. (2003). A parameterization of sea-salt aerosol source function for sub- and super-micron particles. *Global Biogeochem. Cycles* 17, 1–7. doi:10.1029/2003gb002079.

Jaeglé, L., Quinn, P. K., Bates, T. S., Alexander, B., and Lin, J. T. (2011). Global distribution of sea salt aerosols: New constraints from in situ and remote sensing observations. *Atmos. Chem. Phys.* 11, 3137–3157. doi: 10.5194/acp-11-3137-2011.

Keene, W. C., Maring, H., Maben, J. R., Kieber, D. J., Pszenny, A. A. P., Dahl, E. E., et al. (2007). Chemical and physical characteristics of nascent aerosols produced by bursting bubbles at a model air-sea interface. *J. Geophys. Res. Atmos.* 112, 1–16. doi: 10.1029/2007JD008464.

Langmann, B., Scannell, C., and O’Dowd, C. (2008). New Directions: Organic matter contribution to marine aerosols and cloud condensation nuclei. *Atmos. Environ.* 42, 7821–7822. doi: 10.1016/j.atmosenv.2008.09.002.

Lapina, K., Heald, C. L., Spracklen, D. V., Arnold, S. R., Allan, J. D., Coe, H., et al. (2011). Investigating organic aerosol loading in the remote marine environment. *Atmos. Chem. Phys.* 11, 8847–8860. doi: 10.5194/acp-11-8847-2011.

Leck, C., and Bigg, E. K. (2005). Biogenic particles in the surface microlayer and overlaying atmosphere in the central Arctic Ocean during summer. *Tellus B Chem. Phys. Meteorol.* 57, 305–316. doi: 10.3402/tellusb.v57i4.16546.

Lewandowska, A. U., Śliwińska-Wilczewska, S., and Woźniczka, D. (2017). Identification of cyanobacteria and microalgae in aerosols of various sizes in the air over the Southern Baltic Sea. *Mar. Pollut. Bull.* 125, 30–38. doi: 10.1016/j.marpolbul.2017.07.064.

Long, M. S., Keene, W. C., Kieber, D. J., Erickson, D. J., and Maring, H. (2011). A sea-state based source function for size- and composition-resolved marine aerosol production. *Atmos. Chem. Phys.* 11, 1203–1216. doi: 10.5194/acp-11-1203-2011.

Marks, R., Kruczalak, K., Jankowska, K., and Michalska, M. (2001). Bacteria and fungi in air over the Gulf of Gdansk and Baltic Sea. *J. Aerosol Sci.* 32, 237–250. doi: 10.1016/S0021-8502(00)00064-1.

Mayol, E., Arrieta, J. M., Jimenez, M. A., Martinez-Asensio, A., Garcias-Bonet, N., Dachs, J., et al. (2017). Long-range transport of airborne microbes over the global tropical and subtropical ocean. *Nat. Commun.* 8, 201. doi: 10.1038/s41467-017-00110-9.

Mayol, E., Jimenez, M. A., Herndl, G. J., Duarte, C. M., and Arrieta, J. M. (2014). Resolving the abundance and air-sea fluxes of airborne microorganisms in the North Atlantic Ocean. *Front. Microbiol.* 5, 557. doi: 10.3389/fmicb.2014.00557.

Mansour, K., Decesari, S., Facchini, M. C., Belosi, F., Paglione, M., Sandrini, S., et al. (2020). Linking Marine Biological Activity to Aerosol Chemical Composition and cloud-Relevant Properties Over the North Atlantic Ocean. *J. Geophys. Res. Atmos.* 125, 1–19. doi: 10.1029/2019JD032246.

Meskhidze, N., Xu, J., Gantt, B., Zhang, Y., Nenes, A., Ghan, S. J., et al. (2011). Global distribution and climate forcing of marine organic aerosol: 1. Model improvements and evaluation. *Atmos. Chem. Phys.* 11, 11689–11705. doi: 10.5194/acp-11-11689-2011.

Neusüß, C., Gnauk, T., Plewka, A., Herrmann, H., and Quinn, P. K. (2002). Carbonaceous aerosol over the Indian Ocean: OC/EC fractions and selected specifications from size-segregated onboard samples. *J. Geophys. Res. Atmos.* 107, 1–13. doi: 10.1029/2001JD000327.

O’Dowd, C. D., Langmann, B., Varghese, S., Scannell, C., Ceburnis, D., and Facchini, M. C. (2008). A combined organic-inorganic sea-spray source function. *Geophys. Res. Lett.* 35, 1–5. doi: 10.1029/2007GL030331.

O’Dowd, C. D., Facchini, M. C., Cavalli, F., Ceburnis, D., Mircea, M., Decesari, S., et al. (2004). Biogenically driven organic contribution to marine aerosol. *Nature* 431, 676–680. doi: 10.1038/nature02970.

Ovadnevaite, J., O’Dowd, C., Dall’Osto, M., Ceburnis, D., Worsnop, D. R., and Berresheim, H. (2011). Detecting high contributions of primary organic matter to marine aerosol: A case study. *Geophys. Res. Lett.* 38, 2–6. doi: 10.1029/2010GL046083.

Rahav, E., Paytan, A., Chien, C. Te, Ovadia, G., Katz, T., and Herut, B. (2016). The Impact of Atmospheric Dry Deposition Associated Microbes on the Southeastern Mediterranean Sea Surface Water following an Intense Dust Storm. *Front. Mar. Sci.* 3, 1–11. doi: 10.3389/fmars.2016.00127.

Roelofs, G. J. (2008). A GCM study of organic matter in marine aerosol and its potential contribution to cloud drop activation. *Atmos. Chem. Phys.* 8, 709–719. doi: 10.5194/acp-8-709-2008.

Russell, L. M., Hawkins, L. N., Frossard, A. A., Quinn, P. K., and Bates, T. S. (2010). Carbohydrate-like composition of submicron atmospheric particles and their production from ocean bubble bursting. *Proc. Natl. Acad. Sci. U. S. A.* 107, 6652–6657. doi: 10.1073/pnas.0908905107.

Sciare, J., Favez, O., Sarda-Estève, R., Oikonomou, K., Cachier, H., and Kazan, V. (2009). Long-term observations of carbonaceous aerosols in the Austral Ocean atmosphere: Evidence of a biogenic marine organic source. *J. Geophys. Res. Atmos.* 114, 1–10. doi: 10.1029/2009JD011998.

Seifried, J. S., Wichels, A., and Gerdts, G. (2015). Spatial distribution of marine airborne bacterial communities. *Microbiologyopen* 4, 475–490. doi: 10.1002/mbo3.253.

Shank, L. M., Howell, S., Clarke, A. D., Freitag, S., Brekhovskikh, V., Kapustin, V., et al. (2012). Organic matter and non-refractory aerosol over the remote Southeast Pacific: Oceanic and combustion sources. *Atmos. Chem. Phys.* 12, 557–576. doi: 10.5194/acp-12-557-2012.

Sharoni, S., Trainic, M., Schatz, D., Lehahn, Y., Flores, M. J., Bidle, K. D., et al. (2015). Infection of phytoplankton by aerosolized marine viruses. *Proc. Natl. Acad. Sci. USA* 112, 6643–6647. doi: 10.1073/pnas.1423667112.

Sherwood, A. R., Wade, R. M., and Conklin, K. Y. (2020). Seasonality of tropical airborne algae: a 16-month study based on high-throughput sequencing in the Hawaiian Islands. *Grana* 00, 1–12. doi: 10.1080/00173134.2020.1738541.

Spracklen, D. V., Arnold, S. R., Sciare, J., Carslaw, K. S., and Pio, C. (2008). Globally significant oceanic source of organic carbon aerosol. *Geophys. Res. Lett.* 35, 1–5. doi: 10.1029/2008GL033359.

Williams, R. M. (1982). A model for the dry deposition of particles to natural water surfaces. *Atmos. Environ.* 16, 1933–1938. doi: 10.1016/0004-6981(82)90464-4.

Uetake, J., Hill, T. C. J., Moore, K. A., DeMott, P. J., Protat, A., and Kreidenweis, S. M. (2020). Airborne bacteria confirm the pristine nature of the Southern Ocean boundary layer. *Proc. Natl. Acad. Sci. USA* 117, 13275–13282. doi: 10.1073/pnas.2000134117.

Urbano, R., Palenik, B., Gaston, C. J., and Prather, K. A. (2011). Detection and phylogenetic analysis of coastal bioaerosols using culture dependent and independent techniques. *Biogeosciences* 8, 301–309. doi: 10.5194/bg-8-301-2011.

Vignati, E., Facchini, M. C., Rinaldi, M., Scannell, C., Ceburnis, D., Sciare, J., et al. (2010). Global scale emission and distribution of sea-spray aerosol: Sea-salt and organic enrichment. *Atmos. Environ.* 44, 670–677. doi: 10.1016/j.atmosenv.2009.11.013.

Xia, X., Wang, J., Ji, J., Zhang, J., Chen, L., and Zhang, R. (2015). Bacterial Communities in Marine Aerosols Revealed by 454 Pryrosequencing of the 16S rRNA Gene. *J. Atmos. Sci.* 72, 2997–3008. doi: 10.1175/JAS-D-15-0008.1.

Yoon, Y. J., Ceburnis, D., Cavalli, F., Jourdan, O., Putaud, J. P., Facchini, M. C., et al. (2007). Seasonal characteristics of the physicochemical properties of North Atlantic marine atmospheric aerosols. *J. Geophys. Res. Atmos.* 112. doi: 10.1029/2005JD007044.
